# Reidentification of Participants in Shared Clinical Data Sets: Experimental Study

**DOI:** 10.2196/52054

**Published:** 2024-03-15

**Authors:** Daniela Wiepert, Bradley A Malin, Joseph R Duffy, Rene L Utianski, John L Stricker, David T Jones, Hugo Botha

**Affiliations:** 1 Department of Neurology Mayo Clinic Rochester, MN United States; 2 Department of Biomedical Informatics Vanderbilt University Medical Center Nashville, TN United States; 3 Department of Biostatistics Vanderbilt University Medical Center Nashville, TN United States; 4 Department of Computer Science Vanderbilt University Medical Center Nashville, TN United States

**Keywords:** reidentification, privacy, adversarial attack, health care, speech disorders, voiceprint

## Abstract

**Background:**

Large curated data sets are required to leverage speech-based tools in health care. These are costly to produce, resulting in increased interest in data sharing. As speech can potentially identify speakers (ie, voiceprints), sharing recordings raises privacy concerns. This is especially relevant when working with patient data protected under the Health Insurance Portability and Accountability Act.

**Objective:**

We aimed to determine the reidentification risk for speech recordings, without reference to demographics or metadata, in clinical data sets considering both the size of the *search space* (ie, the number of comparisons that must be considered when reidentifying) and the nature of the speech recording (ie, the type of speech task).

**Methods:**

Using a state-of-the-art speaker identification model, we modeled an adversarial attack scenario in which an adversary uses a large data set of identified speech (hereafter, the *known* set) to reidentify as many unknown speakers in a shared data set (hereafter, the *unknown* set) as possible. We first considered the effect of search space size by attempting reidentification with various sizes of known and unknown sets using VoxCeleb, a data set with recordings of natural, connected speech from >7000 healthy speakers. We then repeated these tests with different types of recordings in each set to examine whether the nature of a speech recording influences reidentification risk. For these tests, we used our clinical data set composed of recordings of elicited speech tasks from 941 speakers.

**Results:**

We found that the risk was inversely related to the number of comparisons an adversary must consider (ie, the search space), with a positive linear correlation between the number of false acceptances (FAs) and the number of comparisons (*r*=0.69; P<.001). The true acceptances (TAs) stayed relatively stable, and the ratio between FAs and TAs rose from 0.02 at 1 × 10^5^ comparisons to 1.41 at 6 × 10^6^ comparisons, with a near 1:1 ratio at the midpoint of 3 × 10^6^ comparisons. In effect, risk was high for a small search space but dropped as the search space grew. We also found that the nature of a speech recording influenced reidentification risk, with nonconnected speech (eg, vowel prolongation: FA/TA=98.5; alternating motion rate: FA/TA=8) being harder to identify than connected speech (eg, sentence repetition: FA/TA=0.54) in cross-task conditions. The inverse was mostly true in within-task conditions, with the FA/TA ratio for vowel prolongation and alternating motion rate dropping to 0.39 and 1.17, respectively.

**Conclusions:**

Our findings suggest that speaker identification models can be used to reidentify participants in specific circumstances, but in practice, the reidentification risk appears small. The variation in risk due to search space size and type of speech task provides actionable recommendations to further increase participant privacy and considerations for policy regarding public release of speech recordings.

## Introduction

### Background

Advances in machine learning and acoustic signal processing, along with widely available analysis software and computational resources, have resulted in an increase in voice- and speech-based (hereafter referred to as speech for simplicity) diagnostic and prognostic tools in health care [1]. Applications of such technology range from the early detection of cardiovascular [2], respiratory [3], and neurological [4] diseases to the prediction of disease severity [5] and evaluation of response to treatment [6]. These advances have substantial potential to enhance patient care within neurology given the global burden of neurological diseases [7,8], the poor global access to neurological expertise [9,10], and the established role of speech examination within the fields of neurology and speech-language pathology [11].

Large curated data sets are needed to harness the advances in this area. These data sets are costly to assemble and require rare domain expertise to annotate, leading to increased interest in data sharing among investigators and industry partners. However, given the potentially identifiable nature of voice or speech recordings and the health information contained within such recordings, significant privacy concerns emerge. For many data sets, conventional deidentification approaches that remove identifying metadata (eg, participant demographics and date and location of recording) are sufficient, but sharing speech recordings comes with additional risk as the speech signal itself has the potential to act as a personal identifier [12-14]. In recognition of this potential problem, voiceprints are specifically mentioned as an example of biometric identifiers with respect to the Health Insurance Portability and Accountability Act (HIPAA) Privacy Rule [15,16]. Approaches that involve modifying nonlinguistic aspects of speech through distortion or alteration of the signal may address the inherent identifiability of the speech signal (ie, its potential as a voiceprint) [13,17], but this is not an option when a central part of speech examination in medicine is to use the acoustic signal to detect subtle nonlinguistic abnormalities indicative of the presence of neurological disease [11,13]. Deidentification in compliance with HIPAA may still be possible under the Expert Determination implementation, whereby the risk of reidentification for unmodified speech recordings is deemed low according to accepted statistical and scientific principles [15,16]. In this respect, various previous studies have investigated the risk of reidentification in research cohort data sets based on demographic or other metadata that may link a participant to their corresponding recordings [18-20], but none have explicitly assessed the inherent risk of the acoustic signal itself. Determining the risk of reidentification for recordings in speech data sets and learning how to best mitigate such risk is necessary for health care institutions to protect patients, research participants, and themselves.

Unfortunately, the same machine learning advances that facilitate the use of speech in health care have also made adversarial attacks, such as deanonymization or reidentification attacks, more feasible. For example, attempting to reidentify a speaker from only a speech recording relies on the mature, well-researched field of speaker identification [21,22]. Studies using speaker identification suggest that the potential for identification from the acoustic signal alone is high [23], although there have been minimal studies in the context of adversarial attacks that may result in potential harm to a speaker [24,25]. Only one previous study has relied on a speaker identification model for reidentification, and the results suggested that the risk was high with a single unknown or unidentified speaker and a moderately small reference set of 250 known or identified speakers [25]. As such, the risk inherent in the acoustic signal, devoid of metadata, is nonzero but relatively unknown, and the feasibility for larger data sets is unexplored.

In addition, these approaches are rarely applied to medical speech data sets [26]. This presents a gap in research as medical speech recordings differ from speech recordings of healthy speakers in a few systematic ways. First, the recordings typically contain speech with abnormalities (ie, speech disorders), which may make reidentification harder as many speech disorders are the result of progressive neurological disease, which causes changes in speech that evolve over months to years [11]. Matching recordings from a time when a speaker was healthy or mildly affected to recordings in which they have a more severe speech disorder may be more difficult [27-29]. Second, the premise of speaker identification is that there are recognizable between-speaker differences tied to identity. However, in a cohort enriched with speech with abnormalities, a substantial proportion of the variance would be tied to the underlying speech disorder as this causes recognizable deviations [11], resulting in speakers sounding less distinct [30]. Finally, medical speech recordings typically contain responses to elicited speech tasks rather than the unstructured connected speech typically used in identification experiments. Some speech task responses do contain connected speech (eg, paragraph reading), but others are very dissimilar (eg, vowel prolongation). The impact of speech task on identifiability remains unknown.

### Objectives

In this study, we addressed the risk of reidentification in a series of experiments exploring the reidentifiability of medical speech recordings without using any metadata. We accomplished this goal by modeling an adversarial attack using a state-of-the-art speaker identification architecture wherein an adversary trains the speaker identification model on publicly available, identified recordings and applies the model to a set of unidentified clinical recordings.

## Methods

### Overview

**Figure 1:**
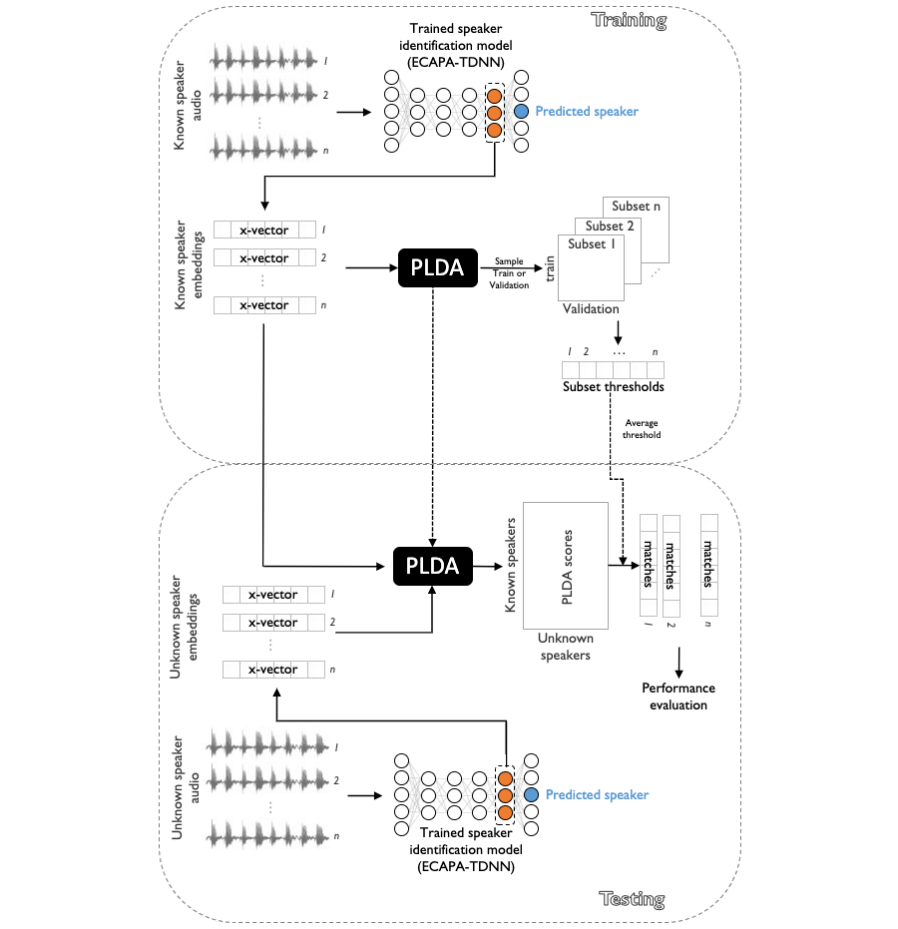
Speaker identification system architecture. During training, recordings from known speakers are fed into a pretrained speaker identification model (ECAPA-TDNN) to extract embeddings. These constitute a low-dimensional, latent representation for each recording that is enriched for speaker-identifying features (x-vectors). We used these x-vectors for known speakers to train a probabilistic linear discriminant analysis (PLDA) classifier and generate an average threshold for acceptance or rejection of a speaker match over several subsets. During testing, the extracted x-vectors are fed into the trained PLDA, and the training threshold is applied, resulting in a set of matches (or no matches) for each recording. ECAPA-TDNN: Emphasized Channel Attention, Propagation, and Aggregation in Time-Delay Neural Network.

Our experimental design was based on the following assumptions: (1) a data recipient has decided to attempt reidentification of study participant data, thereby becoming an adversary; and (2) this adversary relies on an adversarial attack strategy known as a marketer attack, wherein they use a large data set of identified speech (hereafter referred to as the known set), perhaps obtained from a web source such as YouTube, to train a speaker identification model that is then used to reidentify as many unknown speakers in the shared clinical data set (hereafter referred to as the unknown set) as possible [19,31]. Other attack scenarios are possible, but a marketer attack establishes an accepted baseline for risk. To simulate this attack scenario, we built a text-independent speaker identification model with a combination of x-vector extraction using Emphasized Channel Attention, Propagation, and Aggregation in Time-Delay Neural Network (ECAPA-TDNN) [32] and a downstream probabilistic linear discriminant analysis (PLDA)–based classifier [33,34], as described in detail in the following sections. Figure 1 shows the architecture of our model.

### Data

#### Overview

An ideal data set for our attack scenario would consist of (1) a set of elicited speech recordings from tasks typically used in clinical or research speech evaluations and (2) a set of unstructured speech recordings including the same speakers as in item 1 but acquired at a different time and place. This would allow us to directly assess the risk of reidentification of medical recordings by training a model on unstructured connected speech, such as what an adversary may find on the web. Such a data set does not exist. As such, we made use of 2 separate data sets. The first was a combination of the well-known VoxCeleb 1 and 2 data sets, which contain recordings from a web source of >7000 speakers [23,35,36]. The second was a medical speech data set from the Mayo Clinic, which contains recordings of commonly used elicited speech tasks but with fewer speakers.

#### VoxCeleb

The VoxCeleb 1 and 2 data sets are recent large-scale speaker identification data sets containing speech clips extracted from celebrity interviews on YouTube [23,35,36]. The utterances are examples of natural, real-world speech recorded under variable conditions from speakers of different ages, accents, and ethnicities. VoxCeleb 1 and 2 have a combined total of 1,281,762 recordings from 7363 speakers.

#### Mayo Clinic Speech Recordings

The Mayo Clinic clinical speech data set consists of recordings from elicited speech tasks in previously recorded speech assessments. Each speaker has a combination of clips from various tasks commonly used in a clinical speech evaluation, including sentence repetition, word repetition, paragraph reading, alternating motion rates (AMRs), sequential motion rates (SMRs), and vowel prolongation [11]. The clips from speakers vary in recording medium (cassette recording vs DVD), microphone distance, degree of background noise, and presence and severity of motor speech disorder or disorders. There are 19,195 recordings from 941 speakers (the breakdown is presented in Table 1).

**Table 1:** Breakdown of number of recordings and speakers for each task in the Mayo Clinic clinical speech data set.

			Recordings, n (%)		Speakers, n (%)
**Vowel prolongation**					
	“Aaaaaah”	1734 (9.03)		812 (86.3)	
**AMR^a^**					
	“Puh,” “tuh,” and “kuh”	3921 (20.43)		777 (82.6)	
**SMR^b^**					
	“Puh-tuh-kuh”	1049 (5.46)		564 (59.9)	
**Word repetition**					
	“Catapult” and “catastrophe”	124 (0.65)		62^c^ (6.6)	
	Other words	4012^d^ (20.9)		354^c^ (37.6)	
**Sentence repetition**					
	“My physician...”	238 (1.24)		222^e^ (23.6)	
	Other sentences	7505^d^ (39.1)		551^e^ (58.6)	
**Reading passage**					
	“You wish to know...”	612 (3.19)		501 (53.2)	

^a^AMR: alternating motion rate.

^b^SMR: sequential motion rate.

^c^354 total unique speakers.

^d^Samples instead of recordings.

^e^551 total unique speakers.

### X-Vector Extraction Using ECAPA-TDNN

We generated speaker embeddings using a deep neural network to extract fixed-length embedding vectors (x-vectors) from speech recordings [32,34]. This technique has been shown to outperform previous embedding techniques such as i-vectors [37,38] while offering a competitive performance compared to newer end-to-end deep learning approaches [21,22]. Our network of choice was the state-of-the-art ECAPA-TDNN model, which was pretrained on a speaker identification task using VoxCeleb 1 and 2 [32]. This model extracts a 192-dimensional x-vector for each speech recording. The model is publicly available through SpeechBrain, an open-source artificial intelligence speech toolkit [39], and is hosted on Hugging Face.

### PLDA Back-End Classifier

PLDA classifiers are a standard approach for speaker identification due to their ability to reliably extract speaker-specific information from an embedding space using both within- and between-speaker variance [33,40]. PLDA is a dimensionality reduction technique that projects data to a lower-dimensional space where different classes are maximally separated (ie, maximal between-class covariance). The advantage of PLDA over the standard linear discriminant analysis is that it can be generalized to unseen cases [41]. PLDA can then be used to determine whether 2 data points belong to the same class by projecting 2 data points to the latent space and using the distance between them as a measure of similarity. This works well for speaker identification as speaker embeddings are typically fed into a classifier in pairs, where the classifier’s role is to optimally reject or accept the hypothesis that the 2 recordings are from the same speaker. PLDA typically uses the log-likelihood ratio (probability of recordings belonging to the same class vs different classes) to measure similarity, commonly referred to as PLDA scores. During training of a PLDA classifier, PLDA scores for each pairwise comparison in the training set are computed and then used to set a threshold for determining potential speaker matches [33,40].

Our classifier was built and trained on a set of x-vectors extracted from either VoxCeleb or Mayo Clinic speech recordings using ECAPA-TDNN functions from SpeechBrain [39]. We aimed to maximize performance by giving the model multiple speech embeddings per speaker during training, each extracted from recordings under different degradation conditions (eg, varying background noise and microphone distances), which were then averaged to create a single speaker embedding [33].

### Threshold Calculation for Acceptance or Rejection

During training, an optimal threshold needs to be determined to classify whether a given PLDA score represents a match, which can then be applied to new, unseen recordings. Matches that pass the threshold are then considered accepted matches. Generally, the equal error rate (EER) is used to select the threshold [21,22,24,33,34]. The use of the EER assumes that the cost of a false acceptance (FA) is the same as a false rejection (FR) such that the optimal threshold is 1, where the FA rate (FAR) equals the FR rate [22]. While this may be feasible for smaller data sets, when there are several million comparisons, the EER often generates many potential matches per speaker. As such, this can overwhelm the model early on and make it difficult for an adversary to find reliable matches. To scale up to large numbers of comparisons, the adversary must make decisions on how to calibrate the threshold calculation, such as penalizing FAs more heavily even if some true acceptances (TAs) are missed. From an adversary’s perspective, it is less costly to miss TAs if the identified accepted cases have a high likelihood of being true. In effect, precision is more important than recall. The detection cost function (equation 1 [42]) captures this well:

minDCF = C_FR_×FR × prior_target_ + C_FA_ × FA × (1 – prior_target_)**(1)**

We take the cost of an FR (C*_FR_*) multiplied by the total number of FRs and the prior probability of the target and add it to the cost of an FA (C*_FA_*) multiplied by the total number of FAs and the complement of the prior probability.

Using this function, a threshold can be found by setting optimal cost and previous terms based on the adversary’s perspective (ie, avoiding FAs more aggressively) and then finding the FA and FR values that minimize the detection cost function (minDCF) [42]. For example, as the prior probability of the target is lowered (ie, if an adversary expects a small overlap), the calculation puts more emphasis on avoiding FAs (lower FAR) as compared to the EER. Increasing the cost of FAs and decreasing the cost of FRs further prevents FAs.

We used the minDCF with two parameter configurations: (1) the default configuration for the SpeechBrain implementation of the minDCF, where FAs and FRs are penalized equally (*C_FA_*=1; *C_FR_*=1; prior=0.01) [39]; and (2) a strict configuration with a higher penalty for FAs (*C_FA_*=10; *C_FR_*=0.1; prior=0.001).

Due to the large amount of training data in VoxCeleb, it was not computationally feasible to select a threshold for the entire set of identified speakers at once. In addition, we wanted to estimate thresholds that were representative of the population rather than any one subset of speakers. We used a bootstrap sampling technique in which we calculated a minDCF threshold on subsets of training speakers and averaged across runs to estimate the optimal threshold. For each run, latent representations from 2 random subsets of 100 speakers were selected from the training data and fed to the minDCF to calculate a threshold. If the 2 subsets had no overlapping speakers, the entire run was discarded as a threshold could not be calculated. We ran this process between 100 and 500 times depending on the overall number of speakers used for training the PLDA. Training with fewer speakers required fewer runs to converge on an optimal threshold.

### Generating Experimental Speaker Sets

To model the attack scenario, we randomly sampled our data sets to generate the following speaker subsets:

1. *Known set:* this set represents speakers with identified audio data from a web source that the adversary has access to.
2. *Unknown-only set:* this set represents speakers in a shared data set who do not have identifiable audio on the web. No unknown-only speakers are present in the known set.
3. *Overlap set:* this set is a proxy for speakers in a shared data set who do have identifiable audio somewhere on the web. Some speakers from the known set are randomly selected to create this set.
4. *Unknown set:* this represents the full shared data set, consisting of both the unknown-only set and the overlap set.

The number of speakers per set varied based on the experiment. Furthermore, the number of speech recordings per speaker varied between the known and unknown sets. We used all available speech recordings per speaker in the known set but randomly selected only 1 recording per speaker in the unknown set. For overlapping speakers, the selected recording for the unknown set was withheld from the known set. The limit of 1 sample per speaker in the unknown set was based on the nature of a supposed real-world data set where all speech is unlinked and partially deidentified, meaning that the adversary needs to separately find potential matches for each recording even if they come from the same speaker.

Because we randomly subsampled speakers to generate these sets, there is variation in the speakers selected for each experiment, which will result in variability in model performance that is dependent only on the data set. To account for this, we generated multiple speaker splits per experiment. The exact number of splits was dependent on the experiment.

### Experiments

#### VoxCeleb Realistic Experiments: Effect of Search Space Size

We relied on VoxCeleb 1 and 2 to investigate the capability of an attack as a function of the size of the search space (ie, the number of comparisons made to find matching speakers). We reidentified speakers by comparing each speaker in the known set to each speaker in the unknown set. Thus, the search space is the product of the sizes of the known and unknown sets. As such, an increase in either set will increase the number of comparisons. We considered both cases separately, which allowed us to consider one scenario that is dependent on the resources of the adversary (known set size) and another that is under the control of the sharing organization (unknown set size).

To construct a realistic scenario, we assumed that the known and unknown sets would have a low degree of speaker overlap. To justify this assumption, one can consider what would be involved in constructing a set of known speakers. In the absence of metadata about the unknown speakers (eg, the ages and location), there would be no way for an adversary to target a specific population to build their known set. It is unlikely to be feasible for an adversary to manually collect and label speech recordings for a large proportion of the population. Instead, an adversary would likely need to rely on a programmatic approach using easily accessible identifiable audio, such as scraping audio from social media and video- or audio-sharing websites [43]. It is worth noting that this would still be difficult because of several confounding factors: (1) not all members of the population use these websites; (2) not all users have publicly accessible accounts; (3) users with publicly accessible accounts may not have identifiable information linked to them; (4) some accounts post audio or video from multiple speakers, including speakers who also have their own accounts; (5) many users do not post at all; and (6) the population of users is not representative of the general US population, let alone the subset with speech disorders—in terms of the distribution of both age and geographic area [44]. As such, there is no reason to suspect that a patient in a shared medical speech data set would have a high likelihood of existing in an adversary’s set of identified audio recordings.

We also assumed that the adversary would not know which unknown speakers, if any, exist in the known speaker set. Therefore, the adversary must consider all potential matches rather than only focusing on the *N* overall best matches, where *N* is the known overlap. This would reduce the reliability of any match because the likelihood of all potential matches being true is lower than the likelihood of the best *N* matches being true.

We first trained the speaker identification model with the number of speakers in the known set increasing from 1000 to 7205 while maintaining a static unknown set size of 163 speakers, with low speaker overlap between sets (n=5, 3.1% speakers in the overlap set and n=158, 96.9% in the unknown-only set).

We then trained the model with a fixed known set size of 6000 speakers while increasing the number of speakers in the unknown set from 150 to 1000 speakers and maintaining a low overlap of 5 speakers.

Given the low number of overlapping speakers and overall large set sizes, we generated 50 speaker splits for each set size of interest (known set: 1000, 4000, and 7205; unknown set: 150, 500, and 1000).

The acceptance threshold for these experiments was set using the strict minDCF configuration. Experimental parameters are summarized in Table 2.

**Table 2:** Experimental parameters, including number of runs; set sizes; and minimum detection cost function (minDCF) parameters such as the cost of a false acceptance (CFA), cost of a false rejection (CFR), and prior probability (prior).

Experiment	Runs, n	Set size, total speakers				minDCF parameters		
		Known	Unknown	Unknown only	Overlap	C_FA_	C_FR_	Prior
VoxCeleb*:* effect of search space size and known-overlap worst-case scenario	50	1000 to 7205 (varied known) 6000 (varied unknown)	163 (varied known) 150 to 1000 (varied unknown)	158 (varied known) 145 to 995 (varied unknown)	5	10	0.1	0.001
VoxCeleb*:* full-overlap worst-case scenario	20	1000 to 7205 (varied known) 6000 (varied unknown)	163 (varied known) 150 to 1000 (varied unknown)	0	163 (varied known) 150 to 1000 (varied unknown)	10	0.1	0.001
Mayo Clinic speech recordings: cross-task	20	500	55	50	5	1	1	0.01
Mayo Clinic speech recordings: within task	20	500^a^	55	50	5	1	1	0.01

^a^Word repetition: 299 speakers; reading passage: 466 speakers.

#### VoxCeleb Known-Overlap and Full-Overlap Experiments: Worst-Case Scenarios

There are two important initial assumptions in our construction of realistic experiments: (1) the adversary was unaware of the amount of overlap between known and unknown sets, and (2) the amount of overlap was low. Thus, we considered how reidentification risk would be affected if either assumption was incorrect.

First, we considered a potential worst-case scenario in which the adversary did know the number of overlap speakers *N* and, therefore, was able to limit potential matches to the top *N* best matches. As previously mentioned, limiting the number of matches could theoretically improve model reliability, and further reducing the number of matches could produce more noticeable effects. We leveraged our base results from the realistic experiments and only considered the top *N* best matches.

Next, we considered a less realistic worst-case scenario in which all unknown speakers exist in the known speaker set. From an adversary’s perspective, a full-overlap scenario would provide the best chance for them to successfully reidentify speakers because most FAs occur when the model finds a match for unknown speakers who are not in the known speaker set.

We assessed this scenario by replicating the realistic experiments with full overlap between the known and unknown sets. That is, regardless of the unknown set size, all speakers also exist in the known set (no unknown-only set). When increasing the known set size with a fixed unknown set of 163 speakers, the overlap set consists of all 163 speakers, and when increasing the unknown set size with a fixed unknown set, the overlap set is the same as the unknown set size of interest (150, 500, and 1000). In this scenario, we generated only 20 speaker splits for each set size of interest as the larger overlap set led to less variance across runs.

As in the realistic experiments, the acceptance threshold was set using the strict minDCF configuration. Experimental parameters are summarized in Table 2.

#### Mayo Clinic Speech Recording Experiments: Effect of Speech Task

Next, we shifted our focus from the public VoxCeleb data set to a private data set of Mayo Clinic medical speech recordings to look at factors specific to a clinical speech data set, such as whether certain elicited tasks are easier for reidentification and whether being able to link recordings to the same speaker across tasks (pooling) increases risk.

We first compared the performance of the speaker identification model across the various elicited speech tasks in the Mayo Clinic data set based on the same adversarial attack scenario used with the VoxCeleb experiments. In this scenario, the cross-task performance aligns with a real-world case in which the training data contain connected speech recordings (ie, recordings of continuous sequences of sounds such as those of spoken language) but speakers are reidentified using a variety of elicited speech tasks (Table 1). Each task has a different degree of similarity to connected speech (left: most; right: least):

Reading passage > sentence repetition > word repetition > SMR > AMR > vowel prolongation

The reading passage is essentially real-world connected speech in terms of content and duration, but sentence repetition is closer to the connected speech seen in most speech data sets [23]. As such, we selected sentence repetition recordings for speakers in the known set.

The resulting known set comprised 500 speakers and included all sentence repetition recordings, excluding any repetitions of the physician sentence (“My physician wrote out a prescription”), which was saved for the unknown set. We then generated separate unknown sets for each elicited task with 55 speakers (n=5, 9% overlap and n=50, 91% unknown only) who had both sentence repetition recordings and a recording for the given reidentification task (eg, “My physician...” sentence and AMRs).

The known and unknown set sizes were bounded by the number of speakers with sentence repetition recordings (587 speakers) as the sentence-sentence configuration required enough speakers to create a separate known and unknown-only set. We also considered the sentence-sentence configuration (ie, sentence repetitions in both the known and unknown sets) as the realistic baseline.

As a secondary part of this experiment, we pooled all available recordings from all elicited speech tasks (by averaging their embeddings) to generate an unknown set in which the adversary could link recordings from a given speaker (ie, there would be more speech for each unknown speaker).

In addition to the cross-task performance, we compared the within-task performance—where the same elicited speech task is used for both known and unknown speakers—to determine whether anything about the nature of a given speech task affected reidentification. For example, the variance across recordings for the sentence repetition task reflects a combination of static speaker factors (eg, identity and age), dynamic speaker factors (prosody, eg, the same speaker may emphasize different words in a sentence on repeated trials), and content factors (ie, different words in different sentences). In contrast, a task such as AMR involves repeating the same syllable as regularly and rapidly as possible, with most of the variance across speakers likely resulting from static speaker factors. A priori, considering all the elicited tasks, one would expect the proportion of variance across speakers due to dynamic speaker factors to decrease following the same scale as similarity to natural speech. The reading passage would have the most variance due to dynamic speaker factors alone, whereas vowel prolongation would have the least variance. By removing the confounding variable of different elicited tasks for known and unknown speakers (ie, the model is both trained and tested on the same task), we can ascertain whether the qualities of the speech task itself influence reidentification.

We used the same set sizes as the cross-task experiments (500 known, 55 unknown, and 5 overlap) but used recordings from the same elicited speech task in both the known and unknown sets. This setup required at least 2 recordings per speaker for each task. Some tasks had <500 unique speakers or not enough recordings (word repetition and reading passage), so not every known set had exactly 500 speakers. The word repetition task had 299 speakers, and the reading passage task had 466 speakers.

To account for the decrease in the amount of data as compared to the VoxCeleb experiments, we generated only 20 speaker splits per task with default minDCF parameters. Experimental parameters are summarized in Table 2.

### Statistical Analyses

Given that we were simulating an adversarial attack and not optimizing a model, we used random splitting to account for the potential of outlier cases, wherein specific configurations of speakers in the known and unknown sets had a higher-than-average risk of reidentification. We first randomly sampled our larger data set either 20 or 50 times depending on the experiment to generate speaker splits (known, unknown, and overlap sets). We also randomly selected a single recording per speaker in the unknown set to mitigate utterance effects. Furthermore, we used bootstrap sampling of the known (training) set to estimate our acceptance threshold by feeding cohorts of 100 speakers to the minDCF function between 100 and 500 times to converge on an optimal threshold. The exact number of runs was dependent on the overall number of speakers in the known set.

Our primary outcome of interest was the average number of FAs, where the model accepts a match for an unknown speaker without a true match, compared to TAs over several subsampled data sets. Using these counts, we also calculated precision. These metrics informed the reliability of reidentification. Note that TAs and FAs are functionally equivalent to true and false positives, respectively. Using the counts, we also calculated the Pearson correlation coefficient between FAs and set size along with the FAR to determine whether a linear correlation existed between the number of FAs and the number of speakers or comparisons. A 2 tailed *t* test was performed to determine the significance of each correlation.

### Ethical Considerations

The primary data type for this work was clipped speech recordings from either VoxCeleb or our Mayo Clinic clinical speech data set. We could not deidentify the data due to the nature of our work, and the data sets were not anonymous. The VoxCeleb data set has no privacy protections or additional consent processes in place given its public nature—all recordings come from interviews of celebrities posted on YouTube [23,35,36]. For the Mayo Clinic clinical speech data set, we submitted an institutional review board application to the Mayo Clinic to gain permission to use the data. Our work was deemed exempt from additional consent requirements and granted a waiver of HIPAA authorization considering the secondary nature of the analysis. No compensation was offered to participants in the original studies. As the clinical data set may contain private health information, we do not share any recordings or models trained on the clinical recordings. Only researchers at our institution with proper permission can access the clinical data set.

## Results

### VoxCeleb Realistic Experiments: Effect of Search Space Size

**Figure 2:**
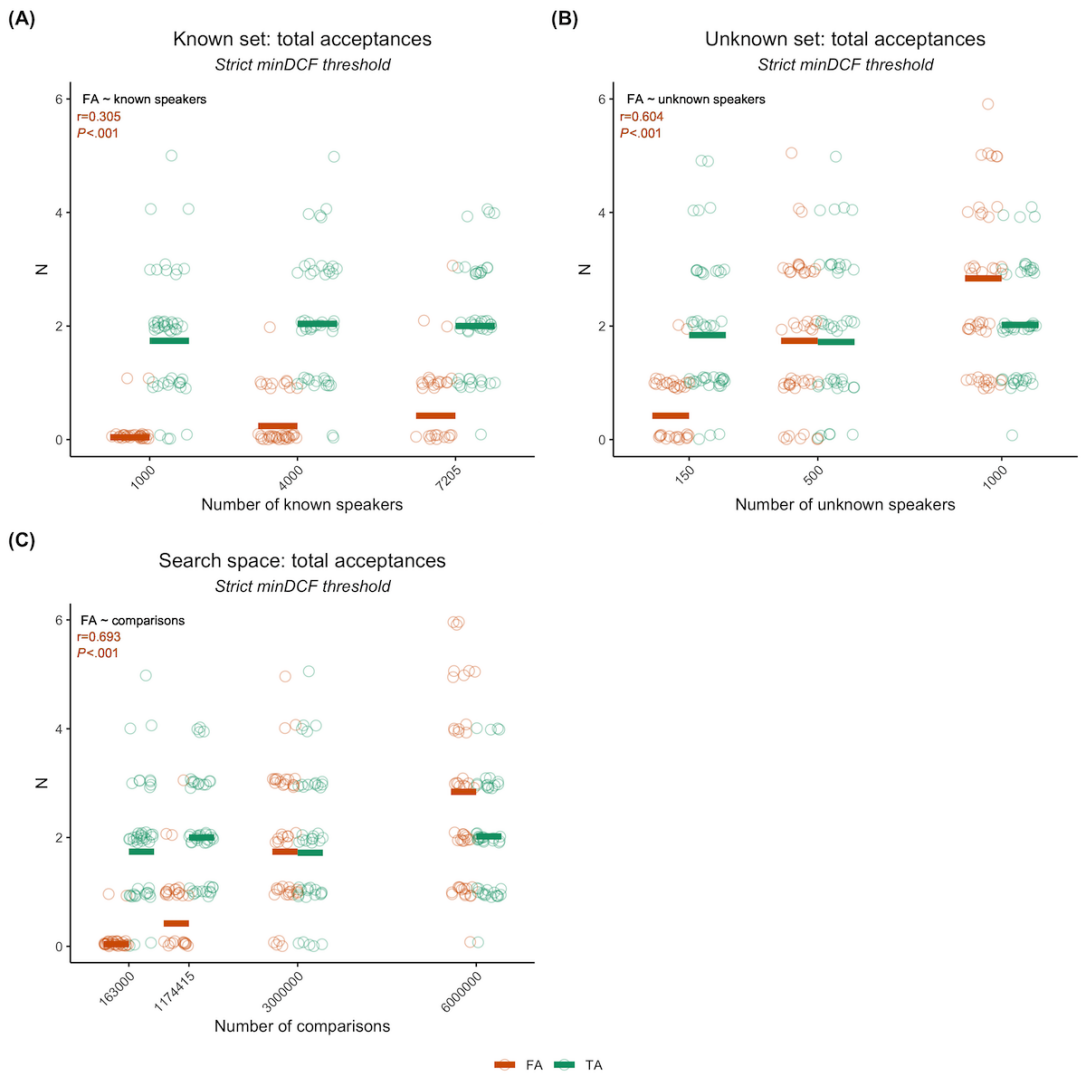
Number of true acceptances (TAs) and false acceptances (FAs) for the speaker recognition model in a realistic scenario using VoxCeleb. (A) shows the counts when varying the number of known speakers while keeping the number of unknown speakers static, (B) shows the counts when varying the number of unknown speakers while keeping the number of known speakers static, and (C) shows the overall trend in terms of the number of comparisons made (ie, the search space size=known × unknown speakers). All plots (A-C) include the Pearson correlation coefficient and corresponding significance for FAs and number of speakers or comparisons. Each run is plotted as a single circle, with red horizontal lines indicating the mean number of FAs and green horizontal lines indicating the mean number of TAs. minDCF: minimum detection cost function.

When training the speaker identification model with increasing numbers of speakers in the known set while maintaining a static unknown set size with low speaker overlap between sets, we found that increasing the number of speakers in the known set resulted in an increase in the mean number of FAs while TAs remained stable, with a linear correlation between FAs and the number of known speakers (*r*=0.30; *P<*.001; *t*_148_=3.89; Figure 2A). Increasing the size of the unknown set had a similar yet more pronounced effect than increasing the known set size, with a higher linear correlation between FAs and the number of unknown speakers (*r*=0.60; *P<*.001; *t*_148_=9.21; Figure 2B).

The difference in effect can be understood based on the geometry of the search space. While the unknown set remains substantially smaller than the known set, adding a speaker to the unknown set will result in a larger increase in the search space than adding a speaker to the known set. As such, we can better demonstrate the overall trend in FAs by considering the results in terms of total comparisons (ie, search space size) rather than individual set size.

**Figure 3:**
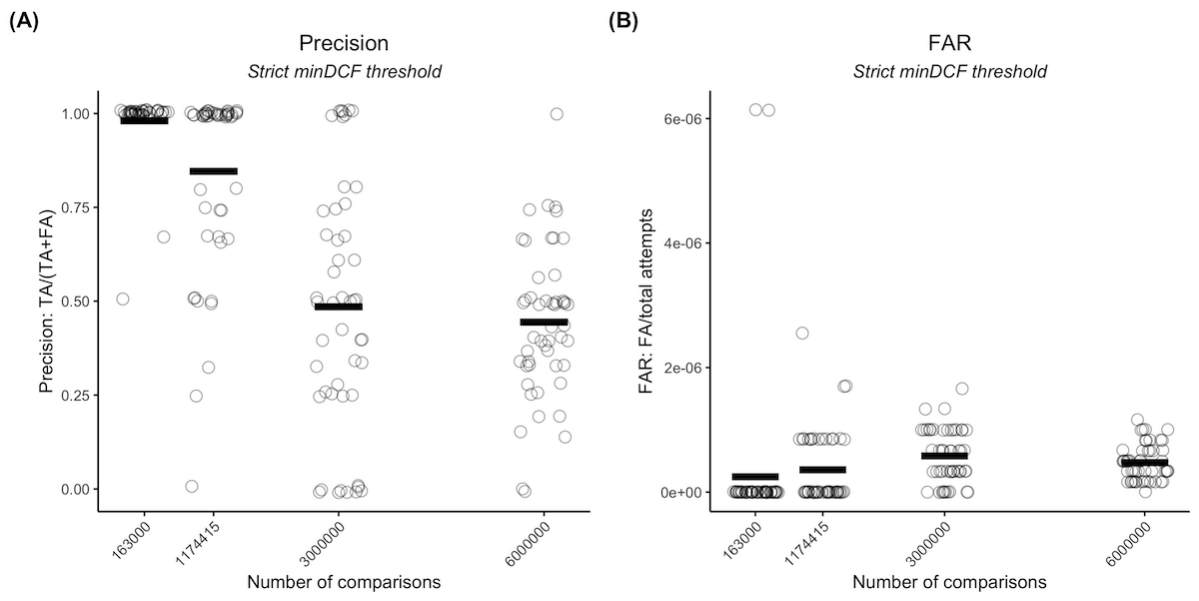
Precision and false acceptance rates (FARs) for the speaker recognition model in a realistic scenario using VoxCeleb. Precision (A) and FARs (B) are shown as a function of the number of comparisons. For both plots, each run is represented by a circle, and the mean is represented by a horizontal black line. FA: false acceptance; minDCF: minimum detection cost function; TA: true acceptance.

We observed that there was a high positive linear correlation between FAs and the number of comparisons (*r*=0.69; *P<*.001; *t*_198_=13.54; Figure 2C), with the mean FAs increasing from 0.04 to 2.84 while TAs remained stable. The ratio between FA and TA (FA/TA) rose from 0.02 at 1 × 10^5^ comparisons to 1.41 at 6 × 10^6^ comparisons, with a near 1:1 ratio at the midpoint of 3 × 10^6^ comparisons. There was a corresponding drop in precision (Figure 3A). It was notable that the FAR remained low and relatively stable, averaging at 4.152 × 10^−7^ (SD 7.255 × 10^−7^; Figure 3B), indicating that the demonstrated trend should hold for the larger numbers of comparisons that we would expect to see in a real attack.

We further observed that using a stricter threshold for matches resulted in our model selecting only 1 match per speaker. This is functionally the same as limiting matches to only the best potential match for each speaker (rank-1 matches), which is an option for an adversary to increase reliability without knowledge of the amount of overlap.

### VoxCeleb Known-Overlap and Full-Overlap Experiments: Worst-Case Scenarios

**Figure 4:**
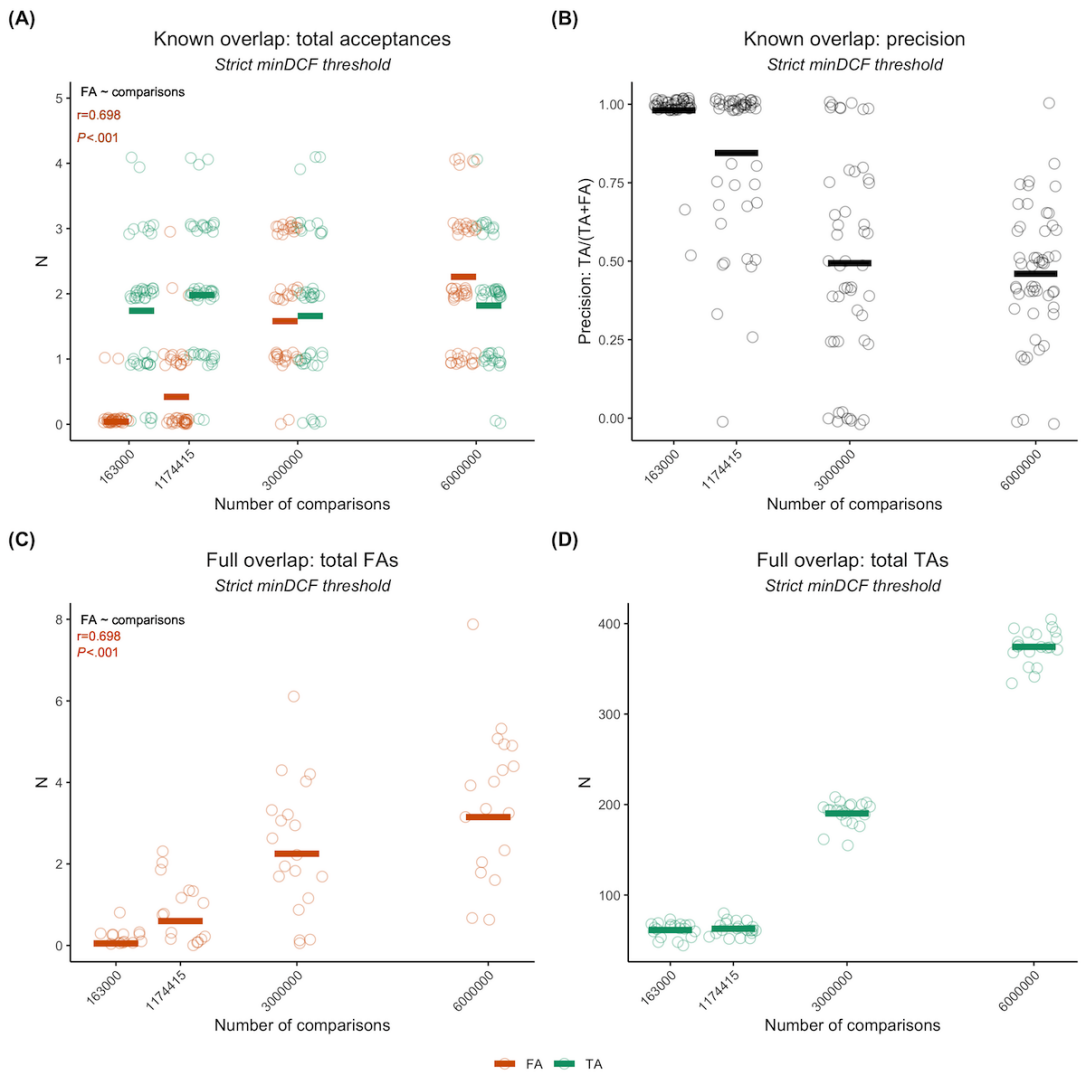
Results for our speaker recognition model in worst-case scenarios using VoxCeleb. (A) shows the true acceptance (TA) and false acceptance (FA) counts for a known-overlap scenario (limited to N=5 best matches), whereas (B) shows the corresponding precision as a function of the number of comparisons (search space size). (C) and (D) show the FA and TA counts for a full-overlap scenario in which all unknown speakers are present in the known speaker set as a function of the number of comparisons (search space size). (A) and (C) also show the Pearson correlation coefficient and corresponding significance between FAs and number of comparisons. Each run is plotted as a single circle, with red horizontal lines indicating the mean number of FAs, green horizontal lines indicating the mean number of TAs, and black horizontal lines indicating the mean precision. minDCF: minimum detection cost function.

When only considering the top *N* best matches, we found that there was still a trend of increasing FAs, with a high linear correlation with the number of comparisons (*r*=0.70; *P<*.001; *t*_198_=13.72; Figure 4A). The FA/TA ratio increased from 0.02 at 1 × 10^5^ comparisons to 1.24 at 6 × 10^6^ comparisons and again had a near 1:1 ratio at 3 × 10^6^ comparisons. These results indicate that some FAs were seen as better matches than some TAs, as further supported by the associated drop in precision (Figure 4B).

When all unknown speakers existed in the known speaker set, the performance improved significantly, with most matches being correct (Figure 4C). Even so, there was still a high positive linear trend for FAs, indicating that, at high overlap, some FAs were ranked higher than TAs (*r*=0.67; *P<*.001; *t*_78_=7.98; Figure 4D). The FA/TA ratio exhibited a fairly large increase considering the number of TAs, increasing from 0.0008 at 1 × 10^5^ comparisons to 0.008 at 6 × 10^6^ comparisons. This is surprising given that, for the realistic experiments, all FAs were associated with matches for nonoverlapping speakers.

### Mayo Clinic Speech Recording Experiments: Effect of Speech Task

**Figure 5:**
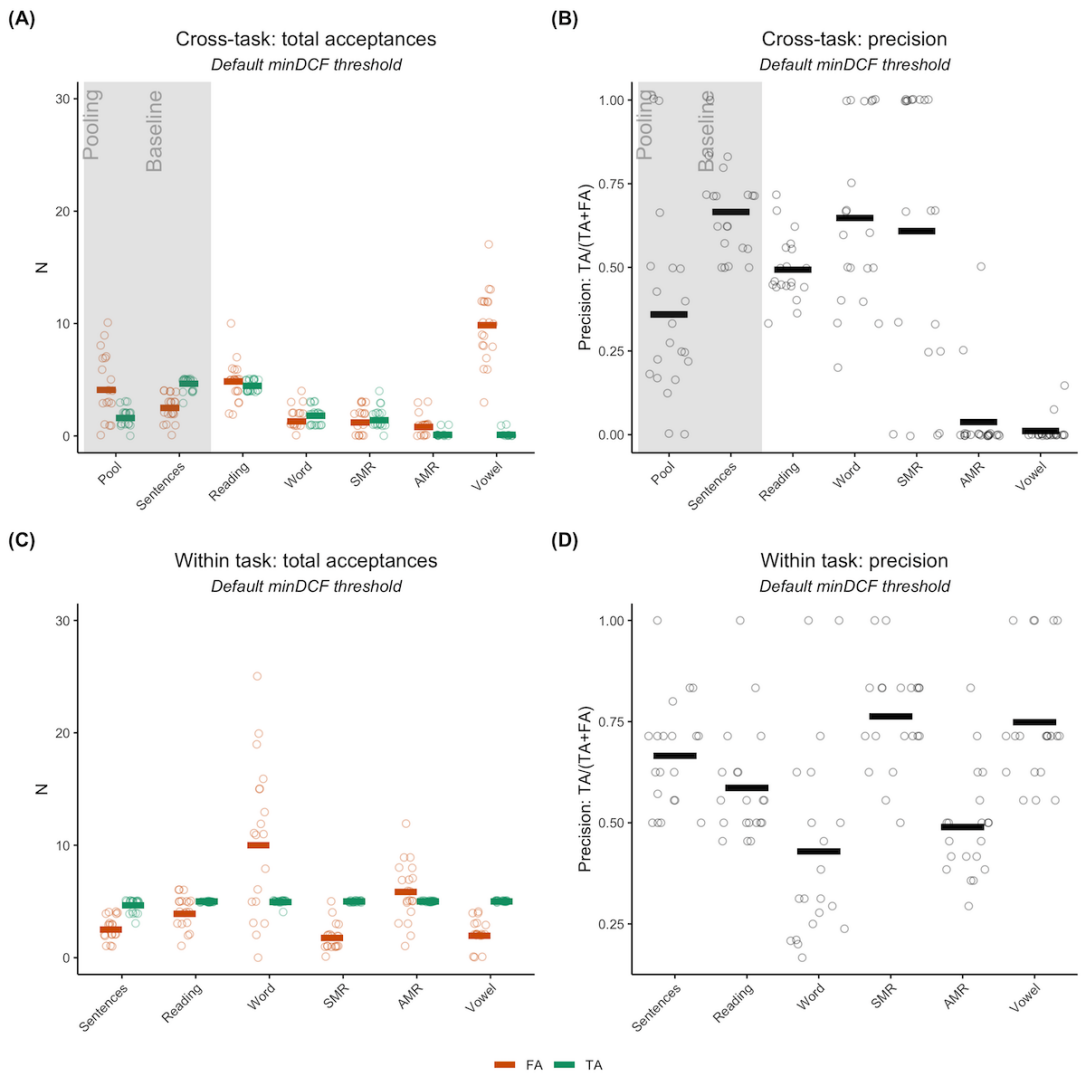
Results for our speaker recognition model using the Mayo Clinic clinical speech data set. (A) and (B) show cross-task results, in which recordings for known speakers are always sentence repetition but the task for unknown speaker recordings varies. The baseline is when sentence repetitions are in both the known and unknown sets. Pooling is when all recordings for an unknown speaker are linked together across all tasks. (A) shows the breakdown of counts for this case, whereas (B) is the corresponding precision. (C) and (D) show within-task results, where tasks for known and unknown speakers are always the same. (C) is the breakdown of counts for this case, whereas (D) is the corresponding precision. Each run is plotted as a single circle, with red horizontal lines indicating the mean number of false acceptances (FAs), green horizontal lines indicating the mean number of true acceptances (TAs), and black horizontal lines indicating the mean precision. AMR: alternating motion rate; minDCF: minimum detection cost function; SMR: sequential motion rate.

We first compared the performance of the speaker identification model across the various elicited speech tasks in the Mayo Clinic data set based on the same adversarial attack scenario used in the VoxCeleb experiments. We observed that the total number of acceptances decreased as the unknown speaker tasks became less similar to the known speaker task, but the proportion of TAs and FAs also varied. This made it more difficult to determine the performance through counts alone (Figure 5A). When considering precision and FA/TA ratio instead, we found that the baseline (sentence-sentence) had the best performance, although the average precision was not high (FA/TA=0.54; precision=66.5%; Figure 5B). The paragraph reading, word repetition, and SMR tasks had a worse performance than the baseline but were comparable to each other in terms of both precision (Figure 5B) and FA/TA ratios (reading passage: FA/TA=1.09; word repetition: FA/TA=0.72; SMR: FA/TA=0.85). However, the AMR and vowel prolongation tasks had extremely low precision and high FA/TA ratios. Vowel prolongation, in particular, had a precision of 0 (almost no TAs across runs) but a high number of FAs, resulting in a ratio of 98.5. Pooling resulted in decreased performance compared to the baseline and the top-performing tasks in terms of both precision (approximately 36%) and FA/TA ratio (2.56). This was likely due to the influence of AMR and vowel prolongation recordings.

The within-task results did not exhibit the same effect as the cross-task results. We found that all tasks reidentified the overlapping speakers (TA=10) but the number of FAs varied drastically across tasks (Figure 5C). Previously, the baseline had the best performance, whereas we instead observed that the SMR and vowel prolongation tasks had the highest precision (Figure 5D), as well as FA/TA ratios of 0.35 and 0.39, respectively. In fact, as tasks became more dissimilar from connected speech and had less variance due to dynamic speaker factors, they saw a relative increase in performance compared to the cross-task scenario. Word repetition was the only exception to this, with lower precision and a greater FA/TA ratio of 2.02 as compared to the cross-task performance.

## Discussion

### Principal Findings

In this study, we investigated the risk of reidentification of unidentified speech recordings without any other speaker- or recording-related metadata. To do so, we performed a series of experiments reflecting a marketer attack by an adversary with access to identified recordings from a large set of speakers and the capability to train a speaker identification model, which would then be used to reidentify unknown speakers in a shared data set. We systematically considered how changes in the size of the data sets and the nature of the speech recordings affected the risk of reidentification. We found that it is feasible to use a speaker identification design—a deep learning speaker embedding extractor (x-vectors) coupled with a PLDA back end—to reidentify speakers in an unknown set of recordings by matching them to recordings from a set of known speakers. Given the performance of current state-of-the-art speaker identification models, this is not surprising. However, these models have only rarely been applied in an adversarial attack scenario [24,25] (ie, their potential as an attack tool for an adversary who aims to reidentify speakers in a shared or publicly available data set was largely unknown). Furthermore, the feasibility of such an attack has not been considered and may have been assumed to be low for speech recordings stripped of all metadata (sometimes referred to as deidentified or anonymous in the literature) without considering the identifiability of the acoustic signal itself [45-48].

Our findings suggest that this is not true. Consistent with a previous study that found a high reidentification risk for an unknown speaker with known sets of up to 250 speakers (search space of ≤250 comparisons) [25], we observed that risk was indeed high for small search spaces. For example, when attempting to reidentify 5 overlapping speakers between a small set of unknown speakers (n=163) and a moderate set of known speakers (n=1000), our model had nearly perfect precision (Figure 3A) and identified 2 speakers on average (FA/TA=0.02; Figure 2A). However, our experiments allowed us to extend this to more realistic search spaces, such as scenarios in which an adversary uses a known speaker set of up to 7205 speakers and an unknown speaker set of up to 1000 speakers (search space of ≤6 million comparisons). We observed that the risk dropped sharply as the search space grew. The FAR was relatively stable at 4.152 × 10^–7^ (Figure 3B), which translates to an average increase of 1 FA for every 2.5 million comparisons. This is a key take-home message from these experiments—increasing the size of the search space, whether by increasing the size of the adversary’s set of identified recordings or of the shared data set, resulted in a corresponding increase in the number of FAs. Given that the number of overlapping speakers remained constant, this suggests that the primary driver of FAs is the size of the nonoverlapping known-to-unknown comparison space (ie, most FAs arise from nonoverlapping unknown speakers being falsely matched to known speakers). In fact, all FAs in the realistic experiments corresponded to nonoverlapping unknown speakers. Here, it is worth noting that, in the experiments in which we only considered the top *N* matches (where *N*=number of overlapping speakers), this trend remained true because some of the FAs scored higher than TAs (Figure 4). This suggests that for a sufficiently large search space, even considering only the best *N* matches will result in many FAs. We pushed this line of reasoning to its limit by considering a worst-case scenario of full overlap in which all unknown speakers had a true match. Even in this scenario, there were still many FAs, and the proportion of FAs increased with increasing search space size. Importantly, this scenario showed that overlapping speakers can still be falsely matched when the overlap is high.

Our experiments with the Mayo Clinic clinical speech recordings allowed us to assess the influence of speech task based on both cross-task and within-task performance. When the model was trained on sentence repetition (ie, the known data set consisted of sentence recordings) and then applied to other tasks (ie, the unknown set consisted of elicited, nonsentence speech), all tasks performed below the baseline, but performance deteriorated most drastically for the less connected speech–like tasks such as AMR and vowel prolongation. These results can be understood with reference to the default minDCF settings, which would penalize FAs and FRs equally. The threshold was chosen using sentence repetition task recordings such that, in most instances, all overlapping speakers were reidentified for unknown sets with connected speech tasks (sentence repetition, paragraph reading, word repetition, and SMRs). The minDCF threshold for these similar tasks resulted in fewer overall acceptances (higher FR rate), but as the tasks diverged from sentence repetition with respect to the degree of connectedness, they were also less likely to be FAs. This suggests that identifiable characteristics learned from training on the sentence repetition task translate well to other connected speech tasks. It also demonstrates the difficulty of choosing a threshold when the tasks in the known set are different from those in the unknown set. Because of the differences within a speaker across tasks, it becomes hard to balance TAs with the flood of FAs as the search space increases. In this instance, a slightly stricter threshold may have been better for the adversary. In contrast, the non–connected speech tasks (AMRs and vowel prolongation) had almost no TAs and a high number of FAs, suggesting that identifiable characteristics from connected speech tasks do not translate to non–connected speech tasks. This is not unexpected given that models perform worse when tested on data that are dissimilar from the training data [49,50]. Following this, we also found that pooling across tasks decreased performance from the baseline. Generally, having more data for a speaker is expected to improve performance, but it is possible that adding recordings of nonsentence tasks to the unknown set hurt performance because the identifiable characteristics are different across tasks and the system is unable to accommodate them. In other words, any helpful characteristics from the connected speech tasks were cancelled out by competing characteristics from the non–connected speech tasks.

In the within-task scenarios, where the known and unknown sets were made up of the same task, the reidentification power for overlapping speakers was better than in the cross-task scenario, but the tasks exhibited vastly different FA rates. In fact, many tasks that were different from connected speech saw improved performance. For example, vowel prolongation, which is nonconnected and the most perceptually different from sentence repetition, exhibited the worst cross-task performance but the second-best within-task performance. This may be because less connected tasks have fewer interfering dynamic speaker factors such that they isolate well the acoustic features that are tied to identity.

Another important finding is that performance for sentence repetition was much weaker than expected based on the VoxCeleb experiments with a larger number of comparisons. We suspect that this may be due to a combination of factors. First, it may be more difficult to differentiate speakers in an unknown set of elicited recordings in which every speaker utters the same sentence. Second, the clinical recordings were all made by patients referred for a speech examination. Consequently, the resulting cohort contained mostly speech with abnormalities, which may impact the PLDA performance. Third, the Mayo Clinic clinical speech data set is smaller than the VoxCeleb data set in terms of both the number of speakers and the number of recordings per speaker, and the recordings are also shorter in duration. This likely had a negative impact on the training of the PLDA classification back end. It remains unknown whether larger clinical data sets or data sets with more recordings per speaker may yield findings more similar to the VoxCeleb results.

Taken together, our findings suggest that the risk of reidentification for a set of clinical speech recordings devoid of any metadata in an attack scenario such as the one we considered in this study is influenced by (1) the number of comparisons that an adversary must consider, which is a function of the size of both the unknown and known data sets; (2) the similarity between the tasks or recordings in the unknown and known data sets; and (3) the characteristics of the recordings in the unknown data set, such as degree of speaker variance and presence and type of speech disorders. These findings translate to actionable goals for both an adversary and the sharing organization.

### Mitigating Privacy Risk

While we assumed that the sharing organization had already reduced risk by stripping recordings of demographic (eg, age or gender) or recording (eg, date or location) metadata, we additionally suggest that reidentification risks could be further reduced by increasing the search space (ie, larger shared data set size) or decreasing the similarity between shared recordings and publicly available recordings (eg, sharing vowel prolongation recordings as long as a publicly available vowel prolongation recording data set does not exist or sharing a larger variety of speech disorder recordings instead of those for a single disorder). Even if the number of overlapping speakers increased with the size of the shared data set, the results from the full-overlap scenario indicate that a model could still have reduced reliability due to an increasing FAR.

In contrast, an adversary can also use this knowledge to enhance their attacks. From their perspective, any additional information that can reduce the search space or increase the similarity between recordings will increase the reliability of speaker matches. This could involve using demographics such as gender, be they shared or predicted by a separate model, to rapidly reduce the number of comparisons. For instance, when the gender balance is 50:50, comparing unknown male individuals to known male individuals would reduce the number of comparisons by 75% (eg, from 6 million to 1.5 million). The adversary may also seek out publicly available recordings of speech with abnormalities to refine their model or models or reduce the search space based on speech disorders. If social media groups exist where identified users with certain medical or speech disorders post videos or audio, an adversary could restrict their known set to these users. Similarly, research participants and support staff may also influence risk through disclosure of participation. By disclosing participation in a study known to share speech recordings, a participant would effectively reduce the size of the known set to 1, increasing their individual risk of reidentification. In addition, having a confirmed match can increase risk overall as the adversary would have a baseline to determine the reliability of matches [51]. Although the focus of this investigation was on the change in relative risk with changes in data set size and speech task, it is worth considering our findings in the context of other factors that impact risk in practice. The most obvious factor is the availability of additional metadata on the speakers or recording. In this respect, it is worth noting that sufficient demographic data, even in the absence of speech, are well known to carry a significant risk of reidentification [19,52]. If any aspect of the metadata makes a patient population unique (ie, there is only one person in a given age range), the risk of reidentification increases [12,14]. Furthermore, the risk is not necessarily the same for all speakers or groups. For example, individuals with rare speech disorders, accents, or other qualities may be easier to match across known and unknown data sets. There may also be identifiable content in the recordings. During less structured speech tasks such as recordings of open-ended conversations, participants may disclose identifiable information about themselves (eg, participants saying where they live). Removing these spoken identifiers is an active area of research [25].

However, it is important to acknowledge that simply because records are vulnerable to reidentification does not mean that they would be reidentified. Notably, when assessing privacy concerns, the probability of reidentification during an attack is conditional on the probability of an attack occurring in the first place [52]. In most instances in which data are shared, the receiving organization or individual will not have any incentive to attempt reidentification. The sharing organization and, in some cases, a receiving organization may also take steps to discourage the risk of an attack. These may take the form of legal (eg, data-sharing agreements) or technical (eg, limited, monitored access) deterrents to a reidentification attack [53]. In contrast, the risk of an attack may be higher for publicly available data sets [54], but there may also be a greater risk of reidentification without a targeted attack. For example, in the field of facial recognition, some companies have scraped billions of photos from publicly available websites to create massive databases with tens of millions of unique faces. These are then used to train a matching algorithm [43], which an end user could query using a photo of an unknown face and obtain a ranked list of matching faces and the source (eg, Facebook). The end user can visit the source website and instantly gain access to other data that may increase or decrease their confidence in a match as well as provide feedback on matches, thereby gradually increasing the performance of the tool as well as the number of known faces. If similar databases are built for speech recordings, they will certainly include publicly available medical speech recordings. Every query to the model would then represent a threat to such a public sample being matched to a queried recording regardless of the intent of the user who queried the model. Such a scenario is difficult to simulate because of the continuously improving nature of the algorithm and the fact that users would incorporate various degrees of nonspeech data.

Refraining from publicly releasing data sets is an obvious mitigation strategy for some of these threats. However, the risk of reidentification must always be balanced with the benefit of data sharing as larger, more representative data sets for the development and testing of digital tools may benefit patients. It is critical that policy makers consider this balance in the context of the rapidly evolving field of artificial intelligence. Naïve approaches such as the “deidentification release-and-forget model” are unlikely to provide sufficient protection [55]. Similarly, informed consent for public release is problematic because the risk of reidentification will be neither static nor easily quantifiable over time. This has led to the development of potential alternative approaches, such as data trusts, synthetic data, federated learning, and secure multiparty computation [56-59].

### Limitations

It should be recognized that there are several notable limitations to our investigation. First, while we relied on state-of-the-art learning architectures, the risk may differ if other computational approaches are considered [21,22]. Second, we did not consider multistage adversarial attacks in which one model is used to predict a demographic, such as sex or age, which is then used to limit the search space, or a scenario in which an adversary manually goes through all potential matches to attempt manual identity verification. However, such approaches would introduce additional uncertainty for the adversary as they would generate predictions for an out-of-sample data set of speech with abnormalities, meaning that accuracy may be lower than expected and the resulting filtered data set may still require many comparisons, in which case our results would apply [60,61]. Third, we did not directly consider the risk of healthy speech versus speech with abnormalities. Nearly all recordings in the Mayo Clinic speech data set contain speech with abnormalities, whereas all VoxCeleb recordings are from healthy speakers. Ideally, there would be a single data set containing both. Fourth, it should be noted that, beyond methodological limitations, our results may not generalize well outside of the United States as the VoxCeleb data have a strong US bias and all the Mayo Clinic recordings were captured in the United States. As such, it will be important to conduct future experiments that leverage alternative computational architectures, more complex adversarial attacks, conversational speech, and data from other geographic regions to assess the reidentification risk for medical speech data more comprehensively.

In addition, there is an important implication of the VoxCeleb experimental design. As we were interested in a range of set sizes and wanted to complete multiple runs for each size, we combined the train and validation sets from VoxCeleb 1 and 2 and randomly selected a holdout set. However, the ECAPA-TDNN model used for extracting embeddings was pretrained on VoxCeleb, meaning it was exposed to most of the recordings (ie, all but the validation cases) during the original training step [32]. The embeddings are almost certainly superior to what one may have obtained if the embedding model was retrained for each of our splits. Unfortunately, that is not a computationally feasible experimental design. Furthermore, superior embeddings mean we are likely to overestimate risk and draw more conservative conclusions. Given the stakes—reidentification of anonymous research patients—we feel this decision was justified. We also ran a set of experiments using the VoxCeleb validation set as our unknown set (Multimedia Appendix 1). This only allowed for a small unknown set with fixed speakers across runs, so it may be overly optimistic regarding risk. In our opinion, the true risk lies in between our main results and the supplementary results.

### Conclusions

IIn summary, our findings suggest that while the acoustic signal alone can be used for reidentification, the practical risk of reidentification for speech recordings, including elicited recordings typically captured as part of a medical speech examination, is low with sufficiently large search spaces. This risk does vary based on the exact size of the search space—which is dependent on the number of speakers in the known and unknown sets—as well as the similarity of the speech tasks in each set. This provides actionable recommendations to further increase participant privacy and considerations for policy regarding the public release of speech recordings. Finally, we also provide ideas for future studies to extend this work, most notably the need to assess other model architectures and data sets as improvements in speaker identification could substantially increase reidentification risk.

### Data Availability

The VoxCeleb 1 and 2 data sets analyzed during this study are available in the VoxCeleb repository [62]. Our Mayo Clinic clinical speech recordings data set analyzed during this study is not publicly available due to the privacy risks related to the release of clinical speech data and are not available by request. We used Python (Python Software Foundation) to implement our code for preprocessing, extracting speaker embeddings, generating subsampled data sets, and running the probabilistic linear discriminant analysis. The source code is available on the internet [63]. The repository also contains detailed documentation for using the scripts.

## Appendix

Multimedia Appendix 1 An additional set of experiments using only the VoxCeleb validation set as the unknown set. This only allowed for a small unknown set with fixed speakers across runs, so it may be overly optimistic regarding risk. These experiments define a lower bound for risk as compared to the original experiments that draw more conservative conclusions and may overestimate risk.

## Abbreviations

AMR: alternating motion rate
ECAPA-TDNN: Emphasized Channel Attention, Propagation, and Aggregation in Time-Delay Neural Network
EER: equal error rate
FA: false acceptance
FAR: false acceptance rate
FR: false rejection
HIPAA: Health Insurance Portability and Accountability Act
minDCF: minimum detection cost function
PLDA: probabilistic linear discriminant analysis
SMR: sequential motion rate
TA: true acceptance
