# Supplementary material for: Reidentification of Participants in Shared Clinical Data Sets: Experimental Study
Source: JMIR AI. 2024 Mar 15;3:e52054. doi: 10.2196/52054 (PMC11041495; doi:10.2196/52054)
Supplement: Multimedia Appendix 1 [file ai_v3i1e52054_app1.docx]

## Supplementary information

The VoxCeleb datasets are originally split into a development set of 7205 speakers and a test set of 163 speakers. In the realistic experiments, we selected speakers randomly from the combined set of VoxCeleb 1 and 2 speakers (7368 total speakers) (see Methods). We also ran a secondary set of experiments where we used only the development set when selecting speakers for the known speaker set and used all the test set as the unknown speaker set. For overlap, we selected 5 random speakers from the known set and added them to the unknown set. As a result of this design, most speakers in the unknown set were fixed across runs.

We found that there were overall more false acceptances (FAs) as compared to the corresponding realistic experiment (Figure 2A), with a higher positive correlation between FAs and number of known speakers (*r* =0*.*78, *P < .*001, *t*_68_ = 10*.*43, Figure S1A versus *r* = 0*.*30, *P < .*001, *t*_148_ = 3*.*89, Figure 2A). As expected, the corresponding precision also dropped much lower (*<* 0*.*25, Figure S1B vs. *>* 0*.*75, Figure 3A). In addition, the average false acceptance rate (FAR) was much higher at 4.993 x 10^−6^ (3.52 x 10^−6^, Figure S1C). As mentioned in the methods, the FAR may have been underestimated in our experimental setup because the ECAPA-TDNN embedding model was exposed to the ‘unknown’ set during its training [32]. This suggests the true FAR for the Voxceleb 1 and 2 datasets likely lie somewhere in between 4.993 x 10^−6^ and 4*.*152 x 10^−7^ (Figure 3B). However, all our conclusions are based on the lower of the FAR bounds, which is the most conservative and privacy promoting option. We present the high end of the FAR bound here to provide further context.


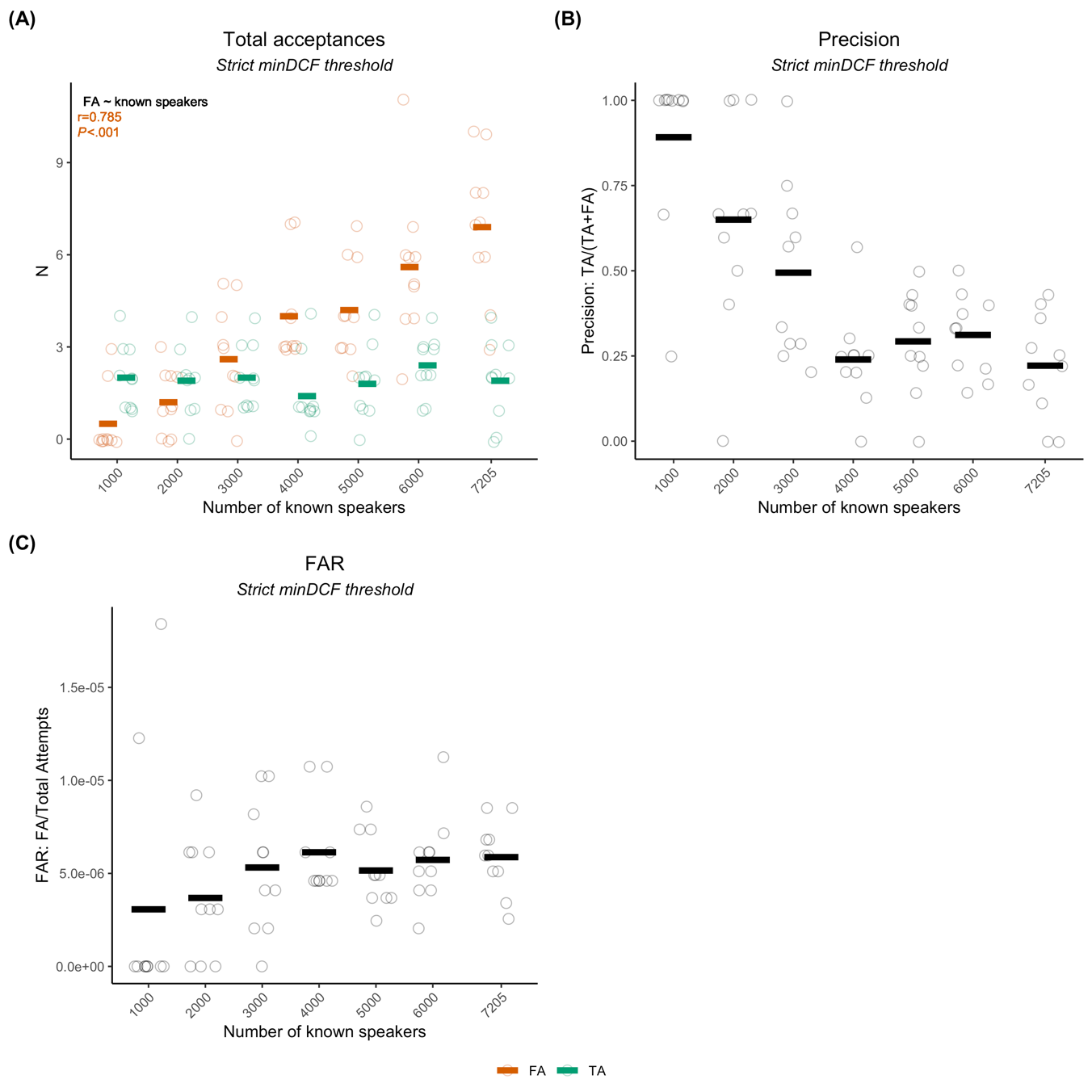


*Figure S1.* *Results for known speaker set with fixed speakers in unknown set using VoxCeleb. (A)* shows the breakdown of true and false acceptances when we change the number of known speakers and keep the unknown speaker set fixed, except for overlapping speakers. It also shows the Pearson’s correlation coefficient and corresponding significance between false acceptances and number of known speakers. *(B)* shows the corresponding precision and *(C)* shows the corresponding false acceptance rates. Each run is plotted as a single circle, with red horizontal lines indicating the mean number of false acceptances, green horizontal lines indicating the mean number of true acceptances, and black horizontal lines indicating the mean precision or false acceptance rate. FA: false acceptance; minDCF: minimum detection cost function; TA: true acceptance.
